# West Nile Virus: An Update Focusing on Southern Europe

**DOI:** 10.3390/microorganisms12122623

**Published:** 2024-12-18

**Authors:** Lara Carrasco, Maria Jose Utrilla, Beatriz Fuentes-Romero, Aitor Fernandez-Novo, Barbara Martin-Maldonado

**Affiliations:** 1 Department of Veterinary Medicine, Biomedical and Health Sciences School, Universidad Europea de Madrid, 28670 Villaviciosa de Odón, Spain; mariajose.utrilla@universidadeuropea.es (M.J.U.); beatriz.fuentes@universidadeuropea.es (B.F.-R.); aitor.fernandez@universidadeuropea.es (A.F.-N.); barbara.martin-maldonado@universidadeuropea.es (B.M.-M.); 2 Veterinary Hospital, Universidad Europea de Madrid, 28670 Villaviciosa de Odón, Spain

**Keywords:** *Flavivirus*, West Nile Fever, arboviral encephalitis, One Health

## Abstract

West Nile Virus (WNV) is a zoonotic, vector-borne pathogen affecting humans and animals, particularly in Europe. The virus is primarily transmitted through mosquitoes that infect birds, which serve as the main reservoirs. Humans and horses are incidental hosts. This review focuses on the epidemiology of WNV in southern Europe, particularly its increasing prevalence. Methods included an extensive literature review and analysis of recent outbreaks. WNV is largely asymptomatic in humans, but a small percentage can develop West Nile neuroinvasive disease (WNND), leading to severe neurological symptoms and fatalities. Horses can also suffer from neurological complications, with high mortality rates. Climate change, migratory birds, and mosquito population dynamics contribute to the virus spread across Europe. Control efforts focus on vector management, and while vaccines are available for horses, none has been approved for humans. Surveillance, particularly of bird and mosquito populations, and further research into the virus molecular structure are crucial for understanding and mitigating future outbreaks.

## 1. Introduction

West Nile Fever (WNF) is an important zoonotic vector-borne disease that can have a great impact on both human and animal health [1]. Its original ecological niche was in the African sylvatic cycle, but since the 1950s, it spread to other continents [2]. It is a single-stranded RNA virus belonging to the family Flaviviridae, genus *Orthoflavivirus*, and classified in the Japanese Encephalitis serocomplex [3]. The first case was a febrile woman reported in Uganda in 1937 [4,5]. Then, in 1962, the first recognized outbreak in Europe occurred in the Camargue region of France, where encephalitis cases were observed in both equids and humans. After 36 years, WNF was reported in the United States of America (USA) in 1999, from where it spread to Canada and South America [6,7]. Nowadays, West Nile Virus (WNV) represents the *Flavivirus* with the broadest geographical distribution [2].

The host range for WNV is extensive, including over 300 species of birds, mammals, reptiles, and amphibians [8]. Wild birds are regarded as the main reservoir of the virus, which is sustained in nature through an enzootic bird–mosquito cycle. The replication of WNV differs across host species, with significantly higher replication rates observed in birds compared to other species [9]. Horses and humans are susceptible to WNV infection, but they are considered dead-end hosts because they do not typically accumulate virus particles at sufficient concentrations to infect mosquitoes [10,11].

Previous research indicates that livestock does not produce sufficient viremia to sustain the virus in endemic regions. However, birds play a crucial role as sylvatic reservoirs and even in spreading viruses to other regions and continents [12]. Although in humans most infections are asymptomatic, neuroinvasive disease and mortality related to WNF have been frequently reported worldwide [1,13]. The number of cases in both humans and animals has significantly increased year by year in the European Union, as has the number of regions where the virus was established as endemic. While the number of confirmed cases in humans was lower in 2023 than in 2022, the number of outbreaks in equids has increased by 51% [14]. Thus, WNV continues to have prominent health relevance as it is considered one of the leading causes of arboviral encephalitis in humans, with the widest distribution worldwide [15]. The increasing incidence of the virus in temperate countries and the socioeconomic consequences of WNF outbreaks have enhanced research on the epidemiology, treatment, and control of this re-emerging disease in Europe.

This review aims to summarize the relevant information about WNF reported in Europe during the last 10 years. For this purpose, an exhaustive search was carried out on PubMed and Google Scholar websites. The search was focused on three items: “West Nile Virus” (item 1), “hosts” (item 2), and “Europe” (item 3). The different search terms for each item were combined by the Boolean operator “AND”. For item 1, the terms were “West Nile Virus” or “West Nile Fever” or “West Nile Disease” or “WNV” OR “WNF”. For item 2, the terms were “Hosts” or “Human” or “Equid” or “Horse” OR “Bird” OR “Avian”. For item 3, the unique term was “Europe”. All the items were mandatory for the inclusion of studies. The period of publication was limited from January 2015 to June 2024. Inclusion criteria were based on the title, abstract, language (English), and year of publication. The search was performed in July 2024. A total of 217 original articles, short communications, and reviews were obtained. Finally, 88 studies were included, plus the last reports from the European Centre for Disease Prevention and Control (ECDC) and other international and national agencies were consulted.

## 2. Update in Phylogenetics and Epidemiology of West Nile Virus

To date, nine different lineages have been proposed that developed from a common ancestor that originated during the XVI century [5,6]. The predictive model presented by Fall et al. [6] suggested that this common ancestor immediately diverged into two strains: one for lineages 1, 5, and 7, and the second for lineages 2, 3, 4, 8, and 9. Nevertheless, other authors suggested that only six lineages are circulating today [16]. Of these, strains 1 and 2 are the most widespread and virulent, responsible for many cases worldwide. WNV lineage 2 was first identified in Europe in 2004 when it was isolated from the brain of a goshawk (*Accipiter gentilis*) in Hungary. Since then, it has spread to various European countries. This lineage is now considered a significant established issue, particularly in countries surrounding the Mediterranean Sea, where the risk of further spread remains high [9] (Table 1).

Many studies have been published over the last years about the genomic structure of WNV and have submitted the sequences of several outbreaks to GenBank, which are the basis of the research in new therapeutics and vaccines [6,17,18]. In this context, the recent description of some G-quadruplex regions in the WNV genome is key for the new effective drug development. These regions of the RNA are characterized by the association of four guanines with significant roles in the regulation of cellular processes. Thus, they could be good targets for new antivirals [18,19].

Other sequencing studies shed light on the complex epidemiology of WNV. For example, a recent publication about the genome sequencing of WNV from mosquitoes and different outbreaks in the Iberian Peninsula suggested the ability of the WNV to overwinter [17]. Moreover, regarding the last outbreaks in southern Europe, all the WNV sequence types seem to come from a common ancestor, although slight genomic differences exist between strains [17,20]. Also, virulence factors and their variations have been identified in different lineages of WNV, such as the pre-membrane protein (prM), the envelope protein (Env), or the helicase protein (NS3) genes [6].

The behavior of the virus has been confirmed to be different between the lineages and between hosts. In vitro studies demonstrated that the WNV strains are very cell-specific. For example, lineage 2 had higher replication in monkey kidney epithelial cells (Vero cells) than in *Aedes pseudocutellaris* cells (Ap61 cells). In contrast, lineage 8 showed a lower replication in mammal cells [6]. In vivo experiments in mice confirmed that lineage 7 was the most virulent, with 100% mortality, in most of the cases without previous clinical signs [6]. In 2017, Pérez-Ramirez et al. [21] assessed the pathogenicity of different WNV strains in mice and classified them into high virulence (lineages 1a, 2, and 7), moderate virulence, and low virulence (1b and 6). Accordingly, the WNV strains isolated from 2017 to 2020 northern goshawks outbreaks in Southern Europe belonged to lineage 2 and showed a moderate-to-high pathogenicity in mice [22].

## 3. Vector Competence and Transmission

The transmission of WNV is mainly through ornithophilous mosquitoes, typically from the *Culex pipiens* complex, although more than 40 mosquito species from the *Culex*, *Aedes*, and *Mansonia* genera can act as vectors [23]. The role of soft ticks of the family Argasidae and Diptera of the genus *Culicoides* as potential vectors of the virus has also been suggested in the arid areas of Africa and southern Russia, although their role as a competent vector is still not well understood [12]. Over 65 species of arthropods have been described as vectors of WNV [6]. In Southern Europe, the *Culex perexiguus* and C. *pipiens* species are particularly significant for WNV’s biological cycle, as they feed on both birds and mammals, closing the bird–mosquito–horse/human transmission cycle [2].

The geographical distribution of the different lineages is influenced by the climatic requirements of the vectors, which are essential for efficient virus replication [24]. The risk of transmission is influenced by environmental temperature and precipitations, which affect the mosquito cycle, increasing the blood feeding and oviposition rate [2,25]. The *Culex* species mentioned above require temperatures between 25 °C and 35 °C to develop optimally, and they enter hibernation when temperatures drop below 10 °C. Conversely, temperatures above 35–40 °C are fatal to these mosquito species [26]. WNF transmission showed a positive correlation with the summer average temperatures, precipitations, and presence of irrigated crops or forests, but negative with the normalized difference water index, which is the water estimated on vegetation and soil [27]. Therefore, in Southern Europe, the periods of highest risk for WNF transmission and development occur at the beginning and end of summer [28]. In this context, in 2022, Cuervo et al. [2] developed a niche model of *C. perexiguus* and *C. pipiens* and confirmed that climatic conditions of all the Iberian Peninsula, except for high-altitude regions, were suitable for the presence of *C. pipiens*. However, *C. perexiguus* had more specific requirements for climatic conditions, so its presence was limited to the basin of two main rivers in Southwestern Spain.

Some authors suggested that mosquitoes are more likely to bite mammals during the last weeks of summer, due to a significant reduction in bird populations. This is when non-resident birds begin migration to wintering areas, leading mosquitoes to become less selective in their feeding [29]. Until now, most WNF outbreaks have been associated with migratory birds, which introduced the virus in Europe, while a few outbreaks have been confirmed as independent introductions [3]. However, as we mentioned above, some authors supported genomic sequencing indicating that the virus could overwinter in Europe, contrasting with the classical theory [17,22,30]. On the other hand, although the main and majority route of transmission is through vectors, other transmission mechanisms have been described in humans: transplacental, through breastfeeding, blood transfusions, and organ transplants [31].

The exponential rise in international trade and tourism over recent decades has increased the likelihood of spreading both vectors and WNV itself across continents [31]. Additionally, the introduction of passerine species, such as the common sparrow, has contributed to the virus’ spread, as these birds can act as reservoirs in urban and peri-urban cycles [24]. Furthermore, global temperature increases, with the control of other flaviviruses, have left ecological niches available for WNV. Changes in precipitation and humidity associated with climate change also play a critical role by altering mosquito habitats and boosting vector activity [32].

## 4. Trends of West Nile Virus in Europe

Since its discovery, WNV has caused disease in humans and animals globally, except for Antarctica [3]. In Europe, WNV was first reported in 1958 in Albania, where neutralizing antibodies were detected in human sera. The first documented outbreak of WNV occurred in southern France in 1962–1963, leading to cases of West Nile neuroinvasive disease (WNND) in both humans and horses. After this outbreak, no further human WNND cases were reported until 1985 [8]. Now, WNV is endemic in Europe and continues to emerge across multiple countries, with lineages 1 and 2 being primarily responsible for disease outbreaks in humans, equids, and birds [3]. According to the European Centre for Disease Prevention and Control (ECDC), Italy, Greece, and Romania accounted for the majority of locally acquired cases, contributing 39%, 20%, and 18% of the European Union’s total cases, respectively [33].

WNV lineage 1 has been circulating in Europe since 1962, when the first human outbreak was reported in France. However, lineage 2 was endemic in Africa until it spread to Europe in 2004 [12]. As we pointed out above, the introduction of WNV in Europe has been classically attributed to migratory birds [12]. Some countries in the south of Europe are a main migratory corridor for hundreds of species that cross our geography in spring, on their way to their breeding regions, and in autumn, to return to their wintering areas. Thus, these countries, such as Italy or Spain, become a gathering area for millions of migratory and resident birds congregating, especially in wetlands rich in mosquitoes, where the probability of transmission is very high [29,34]. Now, recent studies confirmed the virus’ ability to overwinter in Europe and demonstrated that most reported European outbreaks resulted from a few introductions into the continent [12,17,35].

The wide geographic distribution of this flavivirus and the increasing reported cases are influenced by a complex interplay of socioeconomic and ecological factors that have facilitated its spread to new regions, as evidenced in various European countries in recent years [14,16,36]. Various climatic factors have been linked to the incidence of WNV in Europe, with temperature, precipitation, and the Normalized Difference Vegetation Index as the most relevant [37,38]. Plus, these climatic conditions have favored the circulation of other flaviviruses with zoonotic potential in Europe such as the Usutu virus (USUV) [31]. USUV was first detected in Europe in 2001, and although most human infections are asymptomatic, an increasing number of confirmed encephalitis cases have been associated with USUV infections [39].

## 5. Clinical Outbreaks in Southern Europe

### 5.1. Human Outbreaks

In humans, WNV is the main cause of arboviral encephalitis, and high mortality rates have been associated with the virus in the USA (1800 human deaths until 2024), while in Europe, the rate is significantly lower (about 500 human deaths) [29]. Nevertheless, lineage 2 spread rapidly through Central Europe and the Eastern Mediterranean basin (Italy, Greece, and Cyprus, mainly), where outbreaks of both lineages occur annually [20]. In contrast, in Africa, circulating lineages have a lower impact on human health. Neurological symptoms are less frequent despite 80% of humans testing seropositive for WNV [6,40]. Human WNV infections and fatalities were first reported in Greece in 2010, and by 2021, 1420 cases, including 201 deaths, had been recorded across various regions [9]. In Spain, lineage 1 caused outbreaks in 2007, 2010, and 2016 [3,41,42].

**Figure 1:**
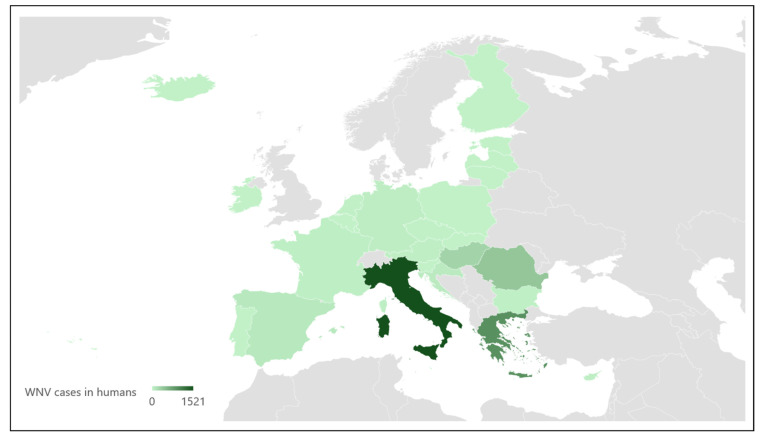
Distribution of West Nile Virus in humans from 2018 to 2022 in Europe (Source: European Food Safety Authority and European Centre for Disease Prevention and Control, 2023 [50]).

During the 2010s, there was a resurgence of clinical cases due to both lineages in the Mediterranean, resulting in 342 human cases with 40 deaths, and a smaller number of horse cases [23,43]. Both lineages now coexist in some European countries; for instance, Italy has experienced annual outbreaks involving both lineages since 2008 [44,45]. Similarly, Cyprus reported the presence of both lineages by 2016 [46], and other nations, including Greece and Serbia, have seen similar patterns. In recent years, the number of reported cases in Europe has increased, with more confirmed cases in 2018 (2083 human cases) than in the entire period between 2010 and 2017 (1832 human cases) [47]. In 2020, 336 cases of locally transmitted WNV disease were reported across Europe, with the majority occurring in Greece, Spain, and Italy [32,48,49]. In the following years, Europe saw 139 human cases reported in 2021, and a significant surge to 1340 cases in 2022, of which only 17 were travel-associated [50]. Specifically, the distribution of WNV human cases confirmed in Europe from 2018 to 2022 is detailed in Figure 1. Moreover, the spread of WNV is wider year by year, and, in 2023, 22 new regions from the European Union reported the infection for the first time [14].

In humans, the viremia peak is observed 4–9 days post-infection, and 80% of infected individuals are asymptomatic [33,34]. For those who develop symptoms, they may take up to 14 days to appear and most commonly resemble flu-like symptoms: fever, headache, fatigue, malaise, myalgia, arthralgia, rash, lymphadenitis, thrombocytopenia, and gastrointestinal signs [33]. The disease generally lasts 2–5 days, with full recovery and lasting immunity (6 months to 1 year) [51]. However, severe WNND cases have been described in 1% of infected individuals, potentially leading to three syndromes: acute flaccid paralysis (5–10% of cases), encephalitis (55–60%), and meningitis (35–40%). These cases have a fatal outcome, especially in immunocompromised or elderly people, resulting in a mortality of about 5–20% of the WNND confirmed cases [5,52]. The risk of developing severe WNND is significantly higher in older individuals. Those over 65 years of age have about a 16 times higher risk of severe disease compared to younger individuals. Furthermore, mortality is about 30 to 45 times greater in individuals over 70 years old compared to younger populations [52]. Moreover, atypical or rare presentations of WNF have been documented, including myocarditis, pancreatitis, hepatitis, cerebellitis, rhabdomyolysis, and ocular manifestations [33,53,54]. The immense variability in clinical outcomes among individuals remains largely unexplained [52]. Plus, 60% of clinically infected patients develop long-term sequelae, such as muscle weakness, fatigue, myalgia, memory loss, depression, and difficulty performing daily activities [55,56]. In 2016, a Belgian research team estimated the economic impact of WNV on human health at more than EUR 47 million, for a virus prevalence of 15%, including hospital charges, work absenteeism, and other related costs [57].

Despite the short-lived viremia, WNV can adhere to red blood cells for several months, so transmission of the virus through blood and organ donations is possible [58,59]. This is of great importance in donors exposed to the virus but asymptomatic. Without a safety protocol for donations, the estimation of WNV-infected blood components in traveling donors exposed to an outbreak was 1 per 100 WNND in Europe. However, in regions with confirmed outbreaks, the risk of an infectious blood product is 113 higher [60]. So, WNV screening in blood donations should be mandatory in countries with demonstrated virus circulation. Also, this screening can be considered a non-biased system of surveillance [59].

### 5.2. Other Mammals’ Outbreaks

WNV is unusual among *Flaviviruses* due to its ability to infect a wide range of hosts. WNV has been detected in 29 species of mammals, which include eastern chipmunks (*Tamias striatus*), striped skunks (*Mephitis mephitis*), fox squirrels (*Sciurus niger*), gray squirrels (*Sciurus carolinensis*), gray wolves (*Canis lupus*), sheep (*Ovis domesticus*), Rocky Mountain goats (*Oreamnos americanus*), big brown bats (*Eptesicus fuscus*), harbor seals (*Phoca vitulina*), domestic cats (*Felis domesticus*), and dogs (*Canis familiaris*). In addition, antibodies against WNV have also been detected in other asymptomatic species including dogs, rodents, wild ungulates, production animals, and zoo animals [1,61,62]. This broad host range contributes to the complex epidemiology of WNV and its ability to persist and spread across different ecosystems [11].

However, most mammalian hosts of WNV are considered inefficient reservoirs because their infective period is relatively short, reducing the chances of mosquitoes becoming infected through bites [11]. Nevertheless, horses are considered accidental hosts and produce insufficient viremia to maintain the biological cycle, but the disease has a great impact on them [6,31]. Despite most infected horses being asymptomatic (70%), mild clinical signs appear in 20% of animals, and the remaining 10% develop neurological signs including mainly ataxia, weakness, and muscle fasciculations [63,64]. Also, other neurological signs have been described with lower frequency: depression, lethargy, partial paralysis, head-pressing, cranial nerve deficits (facial and tongue paralysis), difficulty swallowing, vision disorders, behavior changes, and in the most severe cases, the inability to stand, seizures, and coma. Mortality rates range from 20% to 57% among affected horses [5,12,63,64]. In this context, the economic impact of WNV on horses in Belgium has been estimated at more than EUR 4 million, for a 34% prevalence scenario, including general and replacement costs [57].

One of the first reports of WNV in European horses was in Tuscany, Italy, in September and October 1998, with 38% of seroprevalence at the first sampling [65]. The same virus strain re-emerged in 2008 in Northeastern regions of Italy and caused 33 clinical cases and the death of five horses. When comparing the genome of the envelope protein, the similarity was 100% at the amino acid level [66]. In this context, Italian authorities established a multidisciplinary monitoring program to assess the presence of the virus in humans, horses, birds, and mosquito vectors. This surveillance has been active for more than 15 years, and the annual costs varied from EUR 1.9 to 2.8 million [67]. From 2014 to 2017, WNV prevalence decreased, but in 2018, it rose to 238 confirmed clinical cases [63,67].

In 2023, the number of outbreaks in equids increased by 51% in Europe compared to 2022; seven different countries reported 153 outbreaks. The highest number of confirmed clinical cases in horses was reported by France, Spain, Hungary, and Italy [14]. Moreover, except for Portugal, Austria, and Bulgaria, the rest of the countries also reported human WNV infections in the same region [14]. However, since the WNV notification in equids became mandatory only in 2022, information about the European outbreaks is limited (Table 2).

**Figure 2:**
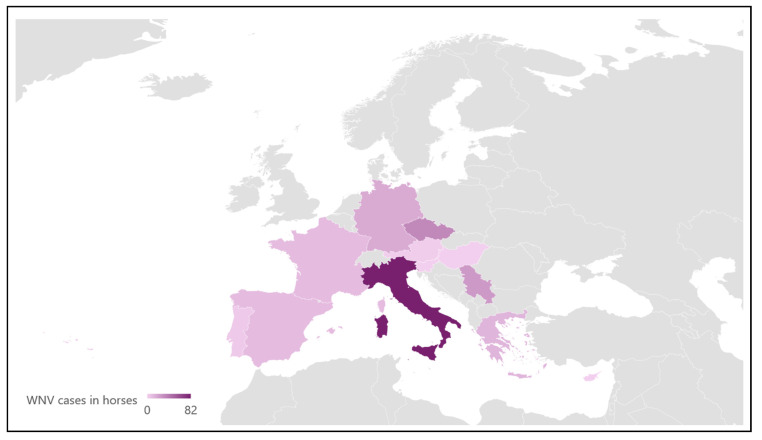
Distribution of West Nile Virus in horses in 2022 in Europe (Source: European Food Safety Authority and European Centre for Disease Prevention and Control, 2023 [50]).

In Spain, 2020 saw an unprecedented WNV epidemic, affecting 139 horse herds [69]. Despite this significant re-emergence of WNV, information on the virus’ circulation within the affected equine herds remains scarce [15]. Until now, the most important WNF wave in horses was in 2018, with 285 outbreaks noticed by the European Centre for Disease Prevention and Control (ECDC) [63]. Notwithstanding, a study carried out in southwestern Spain between 2018 and 2019 confirmed the presence of specific WNV antibodies in almost 20% of the 725 equids sampled, and only 9% of them (13/143) showed neurological signs within the months before sampling [70]. Before that, the surveillance of WNV in Spain from 2010 to 2016 showed that the prevalence of WNV in horses drastically fluctuated from 20.3% in 2010 to 1.7% in 2012 and 40.7% in 2016. In all these cases, the lineage involved was the first one [23]. Figure 2 represents the distribution of WNV in horses from Europe in 2022 noticed in the last European One Health Zoonoses Report published [50].

Among the risk factors, no statistical association has been described between the WNF in horses and the sex or breed of the infected animals [63]. Some authors have reported less seroprevalence in young equids and a higher seroprevalence in individuals with light coat colors than those with dark ones, probably due to mosquitoes’ preferences [70]. Also, the prevalence of WNV has been related to agricultural land use: irrigated lands and rice crops are areas with higher circulating WNV [71]. No studies have been published about WNV transmission through infected blood compounds between animals. However, a case report of a veterinary student being infected with WNV during the necropsy of a WNV-positive horse was reported [72].

### 5.3. Wild Birds’ Outbreaks

Birds are the most important hosts in the WNV life cycle because they can develop viremia levels high enough to infect mosquitoes [8]. Unlike mammals, many bird species develop high concentrations of the virus and remain infectious for extended periods. This prolonged infectivity allows more mosquitoes to become infected, enhancing the virus’ transmission and facilitating its spread. These bird species, particularly those with long viremia periods, play a crucial role in maintaining the WNV cycle in nature. Asymptomatic carriers are particularly significant in the geographical spread of the virus, especially during their annual migrations, as they can transport WNV across regions and introduce it to new areas. This role in long-distance dissemination underscores the importance of birds in the virus’ epidemiology.

Nevertheless, certain bird species are highly susceptible to WNV diseases. These birds can develop severe or fatal encephalitis upon infection [9]. Among the different susceptible avian species, birds of prey have been described as highly susceptible to the virus, probably due to their feeding behavior, and not only migratory species but also resident ones [3,64,73,74,75]. The predation on smaller species also susceptible to WNV, such as amphibians, reptiles, small mammals, and other birds, enhances the probability of acquiring the infection [73]. Some bird species amplify the sylvatic cycle while others contribute to urban and peri-urban cycles, such as the common blackbird (*Turdus merula*), Eurasian collared dove (*Streptopelia decaocto*), and house sparrow (*Passer domesticus*), species abundant and preferred by mosquitoes [24,76]. The Corvidae family (crows, jays, and magpies) has been considered a sentinel species for epidemiological studies and to a lesser extent in raptors, waterfowl, and psittacines [12,31,77].

To date, WNV has caused millions of bird deaths in the USA, particularly affecting more than 300 species, and it has been the main factor for a 45% decline in American crow (*Corvus brachyrhynchos*) populations, with significant environmental impacts [40]. In Europe, bird morbidity and mortality rates are much lower, with fatal cases recorded sporadically [1,74,78]. The first detection of WNV lineage 1 antibodies in wild birds was reported in Greece in 2009 (*Pica pica* and *Corvus cornix*) and lineage 2 in 2010 [79]. From then, WNV has been identified in 10 different wild bird species in this country [80]. Reports on wild birds from Central Europe between 2014 and 2016 confirmed the presence of WNV-specific antibodies at low rates, mainly in raptors and passerines and both resident and migrant species [81]. Recently, in the same region, a survey carried out between 2019 and 2020 included 2345 wild birds from at least 27 species. Among them, 19 birds were positive for the virus, while 80 had antibodies specific against WNV lineage 2. Again, raptors were more prone to infection, and resident and migrant species were involved [75]. In parallel, several confirmed outbreaks have been reported from 2003 to 2020 in birds of prey residents and migrants with neurological signs in Southern Europe [42,82]. Between 2017 and 2020, WNV lineage 2 was detected in four northern goshawks in northeastern Spain [42]. Genetic analyses revealed these isolates were part of the central–southern WNV lineage 2, closely related to the Lombardy cluster identified in Italy in 2013. This cluster has since spread westward, causing outbreaks in France (2018) and Spain (2017, 2020) [22].

**Figure 3:**
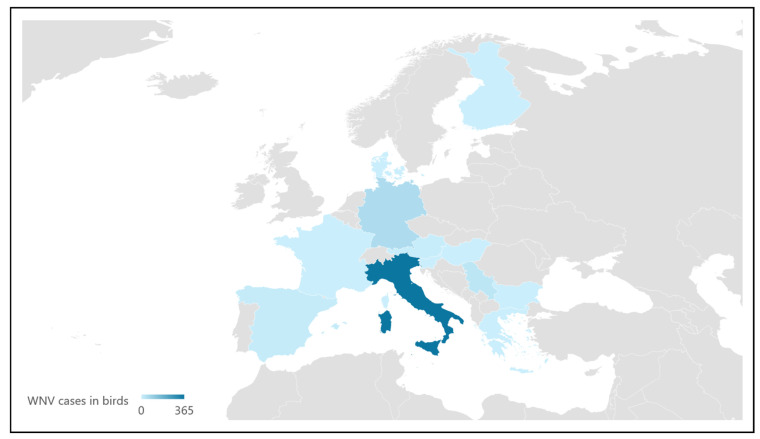
Distribution of West Nile Virus in birds in 2022 in Europe (Source: European Food Safety Authority and European Centre for Disease Prevention and Control, 2023 [50]).

Nevertheless, notification of WNV in wild birds only became mandatory in Europe in 2022, so reliable data are only available for the past two years in European countries (Figure 3). In this sense, in 2023, 251 outbreaks have been reported in eight different countries from the European Union, 22% fewer outbreaks than those reported in 2022 [14]. In wild birds, the disease usually involves high levels of WNV causing severe inflammation, necrosis, and multi-organ hemorrhage, including in the central nervous system. As a *Flavivirus*, WNV readily replicates in primary cell cultures and has a strong affinity for neurons, displaying neurotropic characteristics [5]. Clinical signs include weight loss, reduced daily activity, poor coordination, inability to fly, apathy, lack of response to stimuli, and neurological signs such as torticollis, nystagmus, blindness, opisthotonos, and rhythmic head movements [77,83]. The peak of viremia occurs about 7 days post-infection, and immunity following infection is usually lifelong [84,85]. However, some raptors admitted to different wildlife rescue centers were seropositive to WNV, but none showed specific clinical signs [64,75]. In some cases, their admission was due to traumatism, so a subclinical WNV infection could lead these birds to collisions or other accidents [64]. In this context, the surveillance of wild birds in wildlife rescue centers should be considered as a predictable value of WNV epidemiological trends.

Regarding the risk factors associated with WNV infection in wild birds, a recent study from southwestern Spain reported a statistical difference in the WNV infection between males (48%) and females (3.45%) from the Accipitriformes order, probably due to the behavioral differences in daily dispersion [75]. This contrasts with previous studies in passerines, where no differences were found between sexes [86], and other studies where American robin (*Turdus migratorius*) females were more prone to infection due to the time they spent incubating their eggs and their habitat [87]. Also, statistical differences have been described between adults (>1-year-old) and juveniles (<1-year-old), which could be explained by the daily movements that adults made, while nestlings have been less exposed to mosquitoes, and by the persistence and accumulation of antibodies in adults [42,75,88]. However, the presence of antibodies in juveniles is an indicator of the current WNV circulation [88].

## 6. Comparison of European Situation with Other Regions

Due to the spread over long distances by migratory birds, WNV significantly influences its ecology and global transmission [89]. Lineage 1 shows a complex evolutionary history due to constant connections and genetic flows among countries and continents around the world [90]. In contrast, lineage 2 has a simpler history, with limited introductions from South Africa to Europe, where it became endemic due to favorable eco-climatic conditions [90]. The initial introduction of the virus in Europe established a corridor between Senegal, Morocco, and Western European countries, such as Portugal, Spain, France, and Italy [90,91]. African studies found that *Culex neavei* and *Culex quinquefasciatus* are better transmitters of lineage 1 than lineage 2 [92], while European *Culex pipiens* mosquitoes efficiently transmit both [93]. Factors like mosquito density, feeding preferences, host availability, viral interaction with avian immune systems, and the pathogenicity of strains all contribute to the differing circulation patterns of WNV lineages 1 and 2 between Africa and Europe [94]. Ticks have also been identified as potential vectors of WNV, although their significance remains uncertain. Specifically, WNV isolates have been obtained from the genera *Argas*, *Hyalomma*, *Rhipicephalus*, *Amblyomma*, and *Dermacentor* in Africa [95]. In Europe, the virus has been detected in the same genera plus *Ornithodoros*, as well as in areas from Russia, Azerbaijan, and Moldova [96]. Furthermore, experimental transmission has been observed in *Argas hermanni*, *Ornithodoros capensis*, and *Dermacentor marginatus* [90].

In Africa, bird susceptibility to WNV depends on species and viral strains. While in Europe and America, Passeriformes and Falconiformes are highly vulnerable to WNV lineages 1 and 2, African birds rarely exhibit clinical signs, despite high seroprevalence in regions like Algeria, Egypt, Morocco, and South Africa [90,97,98]. Equids, particularly in Morocco and South Africa, have shown symptomatic infections and fatalities linked to lineages 1 and 2. However, seroprevalence studies confirm WNV circulation in equids across numerous African nations [13,90,99]. Human WNV outbreaks in Africa have been recorded since the 1950s. Cases of neurological symptoms and fatalities associated with WNV-L2 occurred in South Africa, while WNV-L1 has caused encephalitis and deaths in the Mediterranean basin (e.g., Tunisia, Algeria, and Morocco) since 1997. Unfortunately, over 20 African countries lack human WNV seroprevalence studies, underestimating the disease’s burden [90].

WNV arrived in the United States of America (USA) in 1999 and rapidly spread across the continent, causing an epidemic of human disease and massive bird die-offs [7,13]. During this initial outbreak, approximately 8200 individuals were infected in New York City, most of them asymptomatic. However, 20% developed an uncomplicated febrile illness, and only 1/140 individuals experienced the most severe pathological outcomes, such as encephalitis or meningitis [100]. In animals, WNV is also considered a significant pathogen, with more than 28,000 reports in horses and mortal outbreaks in 300 different bird species, resulting in massive population declines [40,101,102]. For example, a decline of 45% has been reported in the American Crow (*Corvus brachyrhynchos*) population following the introduction of WNV [102,103]. Eventually, bird mortality surveillance was integrated with human, equine, and vector surveillance as a tool for monitoring and early detection of virus [104,105,106].

Nevertheless, WNV has not been limited to the USA. More than 5000 human infections have been reported in Canada, and the virus is recognized as an emerging threat across the continent [107]. Although tropical and subtropical regions provide suitable conditions for vectors and WNV establishment (including warm temperatures, diverse avian populations, and a variety of *Culex* mosquitoes), no large outbreaks of WNV have been detected in Latin American countries [108]. This may be due to the presence of other mosquito-borne viruses masking human cases (e.g., misdiagnosed as dengue) or potentially conferring some level of cross-protection [109]. Also, the high biodiversity in these countries can favor the ecological dilution of the virus [110].

## 7. Current Diagnostic Tools

WNF diagnostics are complex. Differential diagnosis should include other *Flaviviruses*, and in equids, also equine herpesvirus myeloencephalopathy, rabies, and bornavirus should be considered, as well as other bacterial, parasitic, or traumatic causes of neurological signs [63].

Several laboratory techniques have been developed to this end, which involve virus isolation, reverse transcription polymerase chain reaction (RT-PCR), serology, and pathological examination [33]. Traditionally, it has been based on serological methods, but the presence of IgM in serum could be due to cross-reactivity with other viruses, like USUV, Japanese encephalitis virus, tick-borne encephalitis virus, or Zika [33,35,59]. Indeed, the ECDC considers cases with a WNV-specific antibody response in serum as probable cases, but not confirmed, according to the Commission Implementing Decision (EU) 2018/945 [111].

Molecular diagnosis, with RT-PCR as the most used for virus detection, is a quick and reliable method that allows for viral genome quantification and screening. However, the low viral load in blood and the short-lived viremia make the diagnosis by molecular techniques difficult [59]. Only in acute infections, blood remains the best sample, as the percentage of virus detection in this period is 86.8%. Several studies confirmed cerebrospinal fluid (CSF) as a better sample to detect WNV, but only for lineages 1 and 2 due to their neuroinvasive character. Also, urine has been described as a more efficient sample than serum, and even CSF, with 58.3% detection in acute infections [59]. The renal excretion of WNV may last for a long period after acute infection in humans, but also in animals [59,112]. In summary, samples for WNV detection by qRT-PCR can be infected tissues, CSF, urine, or blood/serum, in decreasing order of suitability [1,5].

In this context, in Europe, it is mandatory to have the isolation or detection of WNV in blood or cerebrospinal fluid (CSF), the detection of specific IgM in CSF, or the detection of a high titer of WNV-specific IgM in serum AND WNV-specific IgG in serum AND confirmation by neutralization, to confirm positive cases [111]. IgM appears 3–9 days post-infection in CSF and is detectable in 60–70% of human patients with neurological signs. Since IgM cannot cross the blood–brain barrier, its presence in CSF indicates local antibody production by infiltrating lymphocytes [1,5,33].

## 8. New Insights in Treatment and Disease Control

WNV infection provides long-term immunity, often lifelong, and even maternal immunity for several months [5]. Treatment is mainly supportive, with nonsteroidal anti-inflammatory agents. The use of corticosteroids may lead to an increase in viremia. Also, antiviral agents and immunotherapy efficacy have been assessed in humans and animals, with poor results [113]. In humans and horses, recovery can take several months, and it causes frequently the development of permanent neurological sequelae [5]. The recent description of some G-quadruplex regions in the WNV genome is key for the new effective drugs development. These regions of the RNA are characterized by the association of four guanines with significant roles in the regulation of cellular processes. Thus, they could be good targets for new antivirals [18,19]. In mice, Cui et al. [114] successfully demonstrated that the passive transfer of equine WNV-specific antibodies significantly enhanced viral clearance in the spleens and brains of WNV-infected individuals, reducing mortality. Therefore, equine immunoglobulin or equine neutralizing F(ab’)2 passive immunotherapy represents a promising strategy for the prophylactic or therapeutic treatment of patients infected with West Nile Virus [114].

There are no approved vaccines for humans or birds. However, WNV vaccines for horses have been approved in the European Union, with three available options: an inactivated vaccine against lineage 1, an attenuated recombinant vaccine using the Canarypox virus, and an inactivated chimeric vaccine containing prM-E proteins from WNV and yellow fever virus. These vaccines provide cross-immunity between WNV lineages 1 and 2, generate protective antibody levels for at least 6 to 12 months, and safeguard animals from the severe neurologic form of the disease in both field and clinical trials [63]. Consequently, in equids, vaccination is recommended at 5–6 months old, with annual boosters [1,63]. The existing vaccines for WNF in horses are not DIVA, and therefore, it is not possible to differentiate between animals that have been in contact with the virus and vaccinated animals.

For humans and birds, prevention and control measures focus on vector control. Proactive strategies, like applying insecticides (mainly pyrethroids and insect growth regulators) and conducting intensive early-season mosquito control, are preferable (EMCA and WHO, 2013) [115]. However, these measures, especially pesticides and biological controls, can be controversial due to their consequences on wild entomofauna and the environmental risk associated [116]. While there is little evidence of the direct impact of larvicides on WNV outbreak intensity, adulticides seem more effective, and their use has been implemented in several countries depending on national legislation [1,115]. Baz et al. [117] demonstrated the potential of phytochemical compounds as effective, eco-friendly alternatives to synthetic insecticides for mosquito vector management. These plant extracts could provide a safer, biodegradable solution for controlling mosquito populations and reducing disease transmission [117]. Genetic methods for mosquito control offer a promising way to address WNV. The sterilization of mosquito populations using irradiation has been confirmed successfully [118]. However, these techniques face practical, ethical, and regulatory challenges. Despite this, research into genetically engineered mosquitoes, including transgenic approaches, continues due to their potential effectiveness in certain areas [31]. Biological control has been also recommended through different species of fish, invertebrates (e.g., *Copepods*), and microbial agents. Among them, *Bacillus thuringiensis israelensis*, *Lysinibacillus sphaericus*, *Saccharopolyspora spinosa*, *Wolbachia* spp., *Metarhizium anisopliae*, or *Beauveria bassiana*, with appropriate approvals, stand out [118]. Some authors reported the successful application of *Bacillus thuringiensis israelensis*, pyrethroid-based products, and diflubenzuron to control mosquito larval development [119,120]. Additionally, anti-mosquito nets on stable windows, minimizing stagnant waters, and reducing areas prone to mosquito breeding have a significant impact, so awareness campaigns promoting these measures are necessary [1,121]. Specifically, the drainage of channels and large puddles, and the cover of sewage and septic containers in semi-urban and urban areas, have resulted in effective methods for mosquito breeding control [119]. Also, the use of surface treatments to reduce mosquito breeding has been proposed by the World Health Organization [118]. However, there is a need for innovative solutions, such as targeted strategies to reduce vector populations, minimizing the ecological impact at the same [31]. After a systematic review, Odigie et al. [122] found that certain professional groups such as soldiers, veterinarians, agricultural workers, farmers, and laboratory workers were exposed to infected fluids or aerosols that enhance the risk of WNV infection. Identifying these fluids and aerosols is key to improving preventive measures in these professions.

Finally, a robust early warning system requires both passive and active surveillance. In this context, some authors have recommended the implementation of syndromic surveillance, considering that WNV can develop clinical signs in 10% of infected horses, spatially dispersed within human populations, in contrast to the 1% of infected humans [63,123]. Nevertheless, monitoring should not be restricted only to horses and humans, but also it should be implemented in wildlife, particularly, birds and mosquitoes [1,5,41,61].

## 9. Conclusions

In recent years, the regions of Europe with endemic circulation of WNV have been expanding, along with an increase in the number of WNF cases in both humans and animals. However, treatment options remain scarce, and although the disease is self-limiting, a significant percentage of those affected suffer from long-term complications, with a high mortality rate in neuroinvasive cases. As a result, the socioeconomic impact of the disease is considerable. Despite this, no specific effective treatment has been developed, nor has any vaccine been approved for human use. This underscores the need for further research, especially at the molecular level, to identify new targets for specific drugs and to develop effective vaccines. Additionally, given the number of annual outbreaks reported in Europe, there should be ongoing passive surveillance, including routine sampling of horses and wild birds, at a minimum. Due to current limitations, the development of a DIVA vaccine against West Nile Fever in horses is of utmost importance to enhance biosecurity and improve the overall management of West Nile Fever in all populations. The data obtained from this monitoring would provide a deeper understanding of the virus’ epidemiology from a One Health perspective, and the factors that may either promote or reduce the likelihood of infection.

## Figures and Tables

**Table 1:** Distribution and characteristics of the West Nile Virus lineages described to date.

Lineage	Clade	Distribution	Hosts	Observations	References
1	1a	Global	Birds, mammals, and arthropods		[5,7,9,10,11,13,16]
	1b	Oceania	Birds, mammals, and arthropods	Also named *Kunjin* virus	[5,7,9,10,11,13,16]
2		Sub-Saharan Africa, Europe	Birds, mammals, and arthropods		[5,7,8,9,10,11,13,16]
3		Czech Republic	Arthropods	Also named *Rabensburg* virus	[6,8,9]
4	4a	Russia	Amphibians and arthropods	Also named *Krasnodar* virus	[6,8,9]
5		India	Birds, mammals, and arthropods	Potential clade 1c	[6,8,9]
6		Spain	Mosquitoes	Potential clade 4b	[6,8,9]
7		Senegal	Rodents and arthropods	Also named *Koutango* virus	[6,8,9]
8		Senegal			[6,8,9]
9		Austria	Mosquitoes	Potential clade 4c	[6,8,9]

**Table 2:** Clinical outbreaks confirmed in horses from European countries from January 2022 to October 2024 (data available in European Centre for Disease Prevention and Control, ECDC) [14,50,68].

European Countries	Outbreaks in 2022	Outbreaks in 2023	Outbreaks in 2024
Austria	1	1	54
Croatia	0	0	8
Czechia	33	0	0
France	9	44	79
Germany	17	14	174
Greece	12	0	3
Hungary	0	26	41
Italy	82	25	34
Poland	0	0	6
Portugal	3	5	17
Spain	9	38	67
TOTAL	166	153	483

## Data Availability

Not available.

## References

[1] ECDC (European Centre for Disease Prevention and Control) Surveillance, Prevention and Control of West Nile Virus and Usutu Virus Infections in the EU/EEA. https://www.ecdc.europa.eu/en/publications-data/surveillance-prevention-and-control-west-nile-virus-and-usutu-virus-infections.

[2] Cuervo P.F., Artigas P., Mas-Coma S., Bargues M.D. (2021). West Nile virus in Spain: Forecasting the geographical distribution of risky areas with an ecological niche modelling approach. Transbound. Emerg. Dis..

[3] Aguilera-Sepúlveda P., Gómez-Martín B., Agüero M., Jiménez-Clavero M.Á., Fernández-Pinero J. (2021). A new cluster of West Nile virus lineage 1 isolated from a northern goshawk in Spain. Transbound. Emerg. Dis..

[4] Smithburn K.C., Hughes T.P., Burke A.W., Paul J.H. (1940). A neurotropic virus isolated from the blood of a native of Uganda. Am. J. Trop. Med..

[5] Mcvey D.S., Wilson W.C., Gay C.G. (2015). West Nile Virus. Rev. Sci. Tech. OIE.

[6] Fall G., Di Paola N., Faye M., Dia M., De Melo Freire C.C., Loucoubar C., De Andrade Zanotto P.M., Faye O., Sall A.A. (2017). Biological and Phylogenetic Characteristics of West African Lineages of West Nile Virus. PLoS Neglected Trop. Dis..

[7] Hadfield J., Brito A.F., Swetnam D.M., Vogels C.B.F., Tokarz R.E., Andersen K.G., Smith R.C., Bedford T., Grubaugh N.D. (2019). Twenty Years of West Nile Virus Spread and Evolution in the Americas Visualized by Nextstrain. PLoS Pathog..

[8] Chancey C., Grinev A., Volkova E., Rios M. (2015). The Global Ecology and Epidemiology of West Nile Virus. BioMed Res. Int..

[9] Athanasakopoulou Z., Sofia M., Skampardonis V., Giannakopoulos A., Birtsas P., Tsolakos K., Spyrou V., Chatzopoulos D.C., Satra M., Diamantopoulos V. (2023). Indication of West Nile Virus (WNV) Lineage 2 Overwintering among Wild Birds in the Regions of Peloponnese and Western Greece. Vet. Sci..

[10] David S., Abraham A.M. (2016). Epidemiological and Clinical Aspects on West Nile Virus, a Globally Emerging Pathogen. Infect. Dis..

[11] Marra P.P., Grigging S., Caffrey C.L., Kilpatrick A.M., McLean R., Brand C., Saito E.M.I., Dupuis P., Kramer L., Novak R. (2004). West Nile Virus and Wildlife. BioScience.

[12] Sule W.F., Oluwayelu D.O., Hernández-Triana L.M., Fooks A.R., Venter M., Johnson N. (2018). Epidemiology and Ecology of West Nile Virus in Sub-Saharan Africa. Parasites Vectors.

[13] Ronca S.E., Ruff J.C., Murray K.O. (2021). A 20-Year Historical Review of West Nile Virus since Its Initial Emergence in North America: Has West Nile Virus Become a Neglected Tropical Disease?. PLoS Neglected Trop. Dis..

[14] (2023). ECDC (European Centre for Disease Prevention and Control). Epidemiological Update: West Nile Virus Transmission Season in Europe.

[15] Gonzálvez M., Franco J.J., Barbero-Moyano J., Caballero-Gómez J., Ruano M.J., Martínez R., Cano-Terriza D., García-Bocanegra I. (2023). Monitoring the Epidemic of West Nile Virus in Equids in Spain, 2020–2021. Prev. Vet. Med..

[16] Rizzoli A., Bolzoni L., Chadwick E.A., Capelli G., Montarsi F., Grisenti M., De La Puente J.M., Muñoz J., Figuerola J., Soriguer R. (2015). Understanding West Nile Virus Ecology in Europe: *Culex Pipiens* Host Feeding Preference in a Hotspot of Virus Emergence. Parasites Vectors.

[17] Ruiz-López M.J., Muñoz-Chimeno M., Figuerola J., Gavilán A.M., Varona S., Cuesta I., La Puente J.M.-D., Zaballos Á., Molero F., Soriguer R.C. (2023). Genomic Analysis of West Nile Virus Lineage 1 Detected in Mosquitoes during the 2020–2021 Outbreaks in Andalusia, Spain. Viruses.

[18] Terrell J.R., Le T.T., Paul A., Brinton M.A., Wilson W.D., Poon G.M.K., Germann M.W., Siemer J.L. (2024). Structure of an RNA G-Quadruplex from the West Nile Virus Genome. Nat. Commun..

[19] Sarkar S., Armitage B.A. (2021). Targeting a Potential G-Quadruplex Forming Sequence Found in the West Nile Virus Genome by Complementary Gamma-Peptide Nucleic Acid Oligomers. ACS Infect. Dis..

[20] Casimiro-Soriguer C.S., Perez-Florido J., Fernandez-Rueda J.L., Pedrosa-Corral I., Guillot-Sulay V., Lorusso N., Martinez-Gonzalez L.J., Navarro-Marí J.M., Dopazo J., Sanbonmatsu-Gámez S. (2021). Phylogenetic Analysis of the 2020 West Nile Virus (WNV) Outbreak in Andalusia (Spain). Viruses.

[21] Pérez-Ramírez E., Llorente F., Del Amo J., Fall G., Sall A.A., Lubisi A., Lecollinet S., Vázquez A., Jiménez-Clavero M.Á. (2017). Pathogenicity Evaluation of Twelve West Nile Virus Strains Belonging to Four Lineages from Five Continents in a Mouse Model: Discrimination between Three Pathogenicity Categories. J. Gen. Virol..

[22] Aguilera-Sepúlveda P., Napp S., Llorente F., Solano-Manrique C., Molina-López R., Obón E., Solé A., Jiménez-Clavero M.Á., Fernández-Pinero J., Busquets N. (2022). West Nile Virus Lineage 2 Spreads Westwards in Europe and Overwinters in North-Eastern Spain (2017–2020). Viruses.

[23] García-Bocanegra I., Belkhiria J., Napp S., Cano-Terriza D., Jiménez-Ruiz S., Martínez-López B. (2017). Epidemiology and Spatio-Temporal Analysis of West Nile Virus in Horses in Spain between 2010 and 2016. Transbound. Emerg. Dis..

[24] Weaver S.C., Reisen W.K. (2009). Present and Future Arboviral Threats. Antivir. Res..

[25] Ziegler U., Lühken R., Keller M., Cadar D., Van Der Grinten E., Michel F., Albrecht K., Eiden M., Rinder M., Lachmann L. (2018). West Nile Virus Epizootic in Germany, 2018. Antivir. Res..

[26] Moser S.K., Barnard M., Frantz R.M., Spencer J.A., Rodarte K.A., Crooker I.K., Bartlow A.W., Romero-Severson E., Manore C.A. (2023). Scoping Review of Culex Mosquito Life History Trait Heterogeneity in Response to Temperature. Parasites Vectors.

[27] Marcantonio M., Rizzoli A., Metz M., Rosà R., Marini G., Chadwick E., Neteler M. (2015). Identifying the Environmental Conditions Favouring West Nile Virus Outbreaks in Europe. PLoS ONE.

[28] Vogels C.B., Göertz G.P., Pijlman G.P., Koenraadt C.J. (2017). Vector Competence of European Mosquitoes for West Nile Virus. Emerg. Microbes Infect..

[29] Ciota A.T. (2017). West Nile Virus and Its Vectors. Curr. Opin. Insect Sci..

[30] Rudolf I., Betášová L., Blažejová H., Venclíková K., Straková P., Šebesta O., Mendel J., Bakonyi T., Schaffner F., Nowotny N. (2017). West Nile Virus in Overwintering Mosquitoes, Central Europe. Parasites Vectors.

[31] Cendejas P.M., Goodman A.G. (2024). Vaccination and Control Methods of West Nile Virus Infection in Equids and Humans. Vaccines.

[32] Magallanes S., Llorente F., Ruiz-López M.J., La Puente J.M.-D., Soriguer R., Calderon J., Jimenez-Clavero M.Á., Aguilera-Sepúlveda P., Figuerola J. (2023). Long-Term Serological Surveillance for West Nile and Usutu Virus in Horses in South-West Spain. One Health.

[33] Macias A., Martín P., Pérez-Olmeda M., Fernández-Martínez B., Gómez-Barroso D., Fernández E., Ramos J.M., Herrero L., Rodríguez S., Delgado E. (2023). West Nile Virus Emergence in Humans in Extremadura, Spain 2020. Front. Cell. Infect. Microbiol..

[34] Calistri P., Giovannini A., Savini G., Monaco F., Bonfanti L., Ceolin C., Terregino C., Tamba M., Cordioli P., Lelli R. (2009). West Nile Virus Transmission in 2008 in North-Eastern Italy. Zoonoses Public Health.

[35] Emmerich P., Jakupi X., Sherifi K., Dreshaj S., Kalaveshi A., Hemmer C., Hajdari D.P., Von Possel R., Cadar D., Tomazatos A. (2023). Serologic and Genomic Investigation of West Nile Virus in Kosovo. Viruses.

[36] Young J.J., Haussig J.M., Aberle S.W., Pervanidou D., Riccardo F., Sekulić N., Bakonyi T., Gossner C.M. (2021). Epidemiology of Human West Nile Virus Infections in the European Union and European Union Enlargement Countries, 2010 to 2018. Eurosurveillance.

[37] Brugueras S., Fernández-Martínez B., La Puente J.M.-D., Figuerola J., Porro T.M., Rius C., Larrauri A., Gómez-Barroso D. (2020). Environmental Drivers, Climate Change and Emergent Diseases Transmitted by Mosquitoes and Their Vectors in Southern Europe: A Systematic Review. Environ. Res..

[38] Giesen C., Herrador Z., Fernandez-Martinez B., Figuerola J., Gangoso L., Vazquez A., Gómez-Barroso D. (2023). A Systematic Review of Environmental Factors Related to WNV Circulation in European and Mediterranean Countries. One Health.

[39] Vilibic-Cavlek T., Petrovic T., Savic V., Barbic L., Tabain I., Stevanovic V., Klobucar A., Mrzljak A., Ilic M., Bogdanic M. (2020). Epidemiology of Usutu Virus: The European Scenario. Pathogens.

[40] George T.L., Harrigan R.J., LaManna J.A., DeSante D.F., Saracco J.F., Smith T.B. (2015). Persistent Impacts of West Nile Virus on North American Bird Populations. Proc. Natl. Acad. Sci. USA.

[41] López-Ruiz N., Del Carmen Montaño-Remacha M., Durán-Pla E., Pérez-Ruiz M., Navarro-Marí J.M., Salamanca-Rivera C., Miranda B., Oyonarte-Gómez S., Ruiz-Fernández J. (2018). West Nile Virus Outbreak in Humans and Epidemiological Surveillance, West Andalusia, Spain, 2016. Eurosurveillance.

[42] Busquets N., Laranjo-González M., Soler M., Nicolás O., Rivas R., Talavera S., Villalba R., Miguel E.S., Torner N., Aranda C. (2018). Detection of West Nile Virus Lineage 2 in North-Eastern Spain (Catalonia). Transbound. Emerg. Dis..

[43] Napp S., Petrić D., Busquets N. (2018). West Nile Virus and Other Mosquito-Borne Viruses Present in Eastern Europe. Pathog. Glob. Health.

[44] Barzon L., Pacenti M., Franchin E., Lavezzo E., Masi G., Squarzon L., Pagni S., Toppo S., Russo F., Cattai M. (2013). Whole genome sequencing and phylogenetic analysis of West Nile virus lineage 1 and lineage 2 from human cases of infection, Italy, August 2013. Eurosurveillance.

[45] Rizzo C., Napoli C., Venturi G., Pupella S., Lombardini L., Calistri P., Monaco F., Cagarelli R., Angelini P., Bellini R. (2016). West Nile virus transmission: Results from the integrated surveillance system in Italy, 2008 to 2015. Eurosurveillance.

[46] Richter J., Tryfonos C., Tourvas A., Floridou D., Paphitou N.I., Christodoulou C. (2017). Complete Genome sequence of West Nile virus (WNV) from the first human case of neuroinvasive WNV infection in Cyprus. Genome Announc..

[47] Lecollinet S., Pronost S., Coulpier M., Beck C., Gonzalez G., Leblond A., Tritz P. (2019). Viral Equine Encephalitis, a Growing Threat to the Horse Population in Europe?. Viruses.

[48] (2020). ECDC (European Centre for Disease Prevention and Control). Epidemiological Update: West Nile Virus Transmission Season in Europe.

[49] Rodríguez-Alarcón L.G.S.M., Fernández-Martínez B., Moros M.J.S., Vázquez A., Pachés P.J., Villacieros E.G., Martín M.B.G., Borras J.F., Lorusso N., Aceitero J.M.R. (2021). Unprecedented Increase of West Nile Virus Neuroinvasive Disease, Spain, Summer 2020. Eurosurveillance.

[50] (2022). ECDC (European Centre for Disease Prevention and Control). Epidemiological Update: West Nile Virus Transmission Season in Europe.

[51] Barzon L., Pacenti M., Ulbert S., Palù G. (2015). Latest Developments and Challenges in the Diagnosis of Human West Nile Virus Infection. Expert Rev. Anti-Infect. Ther..

[52] Gervais A., Rovida F., Avanzini M.A., Croce S., Marchal A., Lin S.-C., Ferrari A., Thorball C.W., Constant O., Voyer T.L. (2023). Autoantibodies Neutralizing Type I IFNs Underlie West Nile Virus Encephalitis in ∼40% of Patients. J. Exp. Med..

[53] Konjevoda S., Dzelalija B., Canovic S., Pastar Z., Savic V., Tabain I., Barbic L., Peric L., Sabadi D., Stevanovic V. (2019). West Nile Virus Retinitis in a Patient with Neuroinvasive Disease. Rev. Soc. Bras. Med. Trop..

[54] Velasco M., Sánchez-Seco M.P., Campelo C., De Ory F., Martin O., Herrero L., Béliz O.J.S., Minguito T., Campos M.C., Molero F. (2020). Imported Human West Nile Virus Lineage 2 Infection in Spain: Neurological and Gastrointestinal Complications. Viruses.

[55] Carson P.J., Konewko P., Wold K.S., Mariani P., Goli S., Bergloff P., Crosby R.D. (2006). Long-Term Clinical and Neuropsychological Outcomes of West Nile Virus Infection. Clin. Infect. Dis..

[56] Sejvar J.J. (2007). The Long-Term Outcomes of Human West Nile Virus Infection. Clin. Infect. Dis..

[57] Humblet M., Vandeputte S., Fecher-Bourgeois F., Leonard P., Gosset C., Balenghien T., Durand B., Saegerman C. (2016). Estimating the Economic Impact of a Possible Equine and Human Epidemic of West Nile Virus Infection in Belgium. Eurosurveillance.

[58] Piron M., Plasencia A., Fleta-Soriano E., Martinez A., Martinez J.P., Torner N., Sauleda S., Meyerhans A., Escalé J., Trilla A. (2015). Low Seroprevalence of West Nile Virus in Blood Donors from Catalonia, Spain. Vector-Borne Zoonotic Dis..

[59] Lustig Y., Sofer D., Bucris E.D., Mendelson E. (2018). Surveillance and Diagnosis of West Nile Virus in the Face of Flavivirus Cross-Reactivity. Front. Microbiol..

[60] Jimenez R.C.G., Lieshout-Krikke R.W., Janssen M.P. (2021). West Nile Virus and Blood Transfusion Safety: A European Perspective. Vox Sang..

[61] García-Bocanegra I., Paniagua J., Gutiérrez-Guzmán A.V., Lecollinet S., Boadella M., Arenas-Montes A., Cano-Terriza D., Lowenski S., Gortázar C., Höfle U. (2016). Spatio-Temporal Trends and Risk Factors Affecting West Nile Virus and Related Flavivirus Exposure in Spanish Wild Ruminants. BMC Vet. Res..

[62] Garcia-Bocanegra I., Jurado-Tarifa E., Cano-Terriza D., Martinez R., Perez-Marin J.E., Lecollinet S. (2018). Exposure to West Nile Virus and Tick-Borne Encephalitis Virus in Dogs in Spain. Transbound. Emerg. Dis..

[63] Cavalleri J.V., Korbacska-Kutasi O., Leblond A., Paillot R., Pusterla N., Steinmann E., Tomlinson J. (2022). European College of Equine Internal Medicine Consensus Statement on Equine Flaviviridae Infections in Europe. J. Vet. Intern. Med..

[64] Williams R.A.J., Valencia H.A.C., Márquez I.L., González F.G., Llorente F., Jiménez-Clavero M.Á., Busquets N., Barrientos M.M., Ortiz-Díez G., Santiago T.A. (2024). West Nile Virus Seroprevalence in Wild Birds and Equines in Madrid Province, Spain. Vet. Sci..

[65] Autorino G.L., Battisti A., Deubel V., Ferrari G., Forletta R., Giovannini A., Lelli R., Murri S., Scicluna M.T. (2002). West Nile virus epidemic in horses, Tuscany region, Italy. Emerg. Infect. Dis..

[66] Monaco F., Lelli R., Teodori L., Pinoni C., Di Gennaro A., Polci A., Calistri P., Savini G. (2010). Re-emergence of West Nile virus in Italy. Zoonoses Public Health.

[67] Defilippo F., Dottori M., Lelli D., Chiari M., Cereda D., Farioli M., Chianese R., Cerioli M.P., Faccin F., Canziani S. (2022). Assessment of the costs related to West Nile virus monitoring in Lombardy region (Italy) between 2014 and 2018. Int. J. Environ. Res. Public Health.

[68] (2024). ECDC (European Centre for Disease Prevention and Control). Epidemiological Update: West Nile Virus Transmission Season in Europe.

[69] Fiebre del Nilo Occidental. https://www.mapa.gob.es/es/ganaderia/temas/sanidad-animal-higiene-ganadera/sanidad-animal/enfermedades/fiebre-nilo-occidental/F_O_Nilo.aspx.

[70] Guerrero-Carvajal F., Bravo-Barriga D., Martín-Cuervo M., Aguilera-Sepúlveda P., Ferraguti M., Jiménez-Clavero M.Á., Llorente F., Alonso J.M., Frontera E. (2020). Serological Evidence of Co-circulation of West Nile and Usutu Viruses in Equids from Western Spain. Transbound. Emerg. Dis..

[71] García-Carrasco J.-M., Muñoz A.-R., Olivero J., Segura M., García-Bocanegra I., Real R. (2023). West Nile Virus in the Iberian Peninsula: Using Equine Cases to Identify High-Risk Areas for Humans. Eurosurveillance.

[72] Venter M., Steyl J., Human S., Weyer J., Zaayman D., Blumberg L., Leman P.A., Paweska J., Swanepoel R. (2010). Transmission of West Nile Virus during Horse Autopsy. Emerg. Infect. Dis..

[73] Vidaña B., Busquets N., Napp S., Pérez-Ramírez E., Jiménez-Clavero M.Á., Johnson N. (2020). The Role of Birds of Prey in West Nile Virus Epidemiology. Vaccines.

[74] Bravo-Barriga D., Aguilera-Sepúlveda P., Guerrero-Carvajal F., Llorente F., Reina D., Pérez-Martín J.E., Jiménez-Clavero M.Á., Frontera E. (2021). West Nile and Usutu Virus Infections in Wild Birds Admitted to Rehabilitation Centres in Extremadura, Western Spain, 2017–2019. Vet. Microbiol..

[75] Ziegler U., Bergmann F., Fischer D., Müller K., Holicki C.M., Sadeghi B., Sieg M., Keller M., Schwehn R., Reuschel M. (2022). Spread of West Nile Virus and Usutu Virus in the German Bird Population, 2019–2020. Microorganisms.

[76] Figuerola J., Jiménez-Clavero M.Á., Ruíz-López M.J., Llorente F., Ruiz S., Hoefer A., Aguilera-Sepúlveda P., Jiménez-Peñuela J., García-Ruiz O., Herrero L. (2022). A One Health View of the West Nile Virus Outbreak in Andalusia (Spain) in 2020. Emerg. Microbes Infect..

[77] Erdélyi K., Ursu K., Ferenczi E., Szeredi L., Rátz F., Skáre J., Bakonyi T. (2007). Clinical and Pathologic Features of Lineage 2 West Nile Virus Infections in Birds of Prey in Hungary. Vector-Borne Zoonotic Dis..

[78] Gray T., Webb C.E. (2014). A Review of the Epidemiological and Clinical Aspects of West Nile Virus. Int. J. Gen. Med..

[79] Valiakos G., Touloudi A., Athanasiou L.V., Giannakopoulos A., Iacovakis C., Birtsas P., Spyrou V., Dalabiras Z., Petrovska L., Billinis C. (2012). Serological and molecular investigation into the role of wild birds in the epidemiology of West Nile virus in Greece. Virol. J..

[80] Sofia M., Giannakopoulos A., Giantsis I.A., Touloudi A., Birtsas P., Papageorgiou K., Athanasakopoulu Z., Chatzopoulos D.C., Vrioni G., Galamatis D. (2022). West Nile virus occurrence and ecological niche modeling in wild bird species and mosquito vectors: An active surveillance program in the Peloponnese region of Greece. Microorganisms.

[81] Michel F., Fischer D., Eiden M., Fast C., Reuschel M., Müller K., Rinder M., Urbaniak S., Brandes F., Schwehn R. (2018). West Nile Virus and Usutu Virus Monitoring of Wild Birds in Germany. Int. J. Environ. Res. Public Health.

[82] Alba A., Allepuz A., Napp S., Soler M., Selga I., Aranda C., Casal J., Pages N., Hayes E.B., Busquets N. (2013). Ecological Surveillance for West Nile in Catalonia (Spain), Learning from a Five-Year Period of Follow-up. Zoonoses Public Health.

[83] Guía sobre Vigilancia Sanitaria en Fauna Silvestre Ministerio de Agricultura, Pesca y Alimentación (MAPA). https://www.mapa.gob.es/va/ganaderia/temas/sanidad-animal-higiene-ganadera/guiavigilanciasanitariafaunasilvestre_tcm39-511596.PDF.

[84] McLean R.G., Ubico S.R., Bourne D., Komar N. (2002). West Nile Virus in Livestock and Wildlife. Curr. Top. Microbiol. Immunol..

[85] Jiménez-Clavero M.Á. (2012). Animal Viral Diseases and Global Change: Bluetongue and West Nile Fever as Paradigms. Front. Genet..

[86] Medrouh B., Lafri I., Beck C., Leulmi H., Akkou M., Abbad L., Lafri M., Bitam I., Lecollinet S. (2020). First Serological Evidence of West Nile Virus Infection in Wild Birds in Northern Algeria. Comp. Immunol. Microbiol. Infect. Dis..

[87] Egizi A.M., Farajollahi A., Fonseca D.M. (2014). Diverse Host Feeding on Nesting Birds May Limit Early-Season West Nile Virus Amplification. Vector-Borne Zoonotic Dis..

[88] La Puente J.M.-D., Ferraguti M., Ruiz S., Roiz D., Llorente F., Pérez-Ramírez E., Jiménez-Clavero M.Á., Soriguer R., Figuerola J. (2018). Mosquito Community Influences West Nile Virus Seroprevalence in Wild Birds: Implications for the Risk of Spillover into Human Populations. Sci. Rep..

[89] Georgopoulou I., Tsiouris V. (2008). The potential role of migratory birds in the transmission of zoonoses. Vet. Ital..

[90] Mencattelli G., Ndione M.H.D., Rosà R., Marini G., Diagne C.T., Diagne M.M., Fall G., Faye O., Diallo M., Faye O. (2022). Epidemiology of West Nile virus in Africa: An underestimated threat. PLoS Neglected Trop. Dis..

[91] Ndione M.H.D., Ndiaye E.H., Faye M., Diagne M.M., Diallo D., Diallo A., Sall A.A., Loucoubar C., Faye O., Diallo M. (2022). Re-introduction of West Nile virus lineage 1 in Senegal from Europe and subsequent circulation in human and mosquito populations between 2012 and 2021. Viruses.

[92] Fall G., Diallo M., Loucoubar C., Faye O., Sall A.A. (2014). Vector competence of *Culex neavei* and *Culex quinquefasciatus* (Diptera: Culicidae) from Senegal for lineages 1, 2, Koutango and a putative new lineage of West Nile virus. Am. J. Trop. Med. Hyg..

[93] Fros J.J., Geertsema C., Vogels C.B., Roosjen P.P., Failloux A.B., Vlak J.M., Koenraadt C.J., Takken W., Pijlman G.P. (2015). West Nile virus: High transmission rate in north-western European mosquitoes indicates its epidemic potential and warrants increased surveillance. PLoS Neglected Trop. Dis..

[94] Fiacre L., Pagès N., Albina E., Richardson J., Lecollinet S., Gonzalez G. (2020). Molecular determinants of West Nile virus virulence and pathogenesis in vertebrate and invertebrate hosts. Int. J. Mol. Sci..

[95] Lwande O.W., Lutomiah J., Obanda V., Gakuya F., Mutisya J., Mulwa F., Michuki G., Chepkorir E., Fischer A., Venter M. (2013). Isolation of tick and mosquito-borne arboviruses from ticks sampled from livestock and wild animal hosts in Ijara District, Kenya. Vector-Borne Zoonotic Dis..

[96] Hubálek Z., Halouzka J. (1999). West Nile fever--a reemerging mosquito-borne viral disease in Europe. Emerg. Infect. Dis..

[97] Taylor R.M., Work T.H., Hurlbut H.S., Rizk F. (1956). A study of the ecology of West Nile virus in Egypt. Am. J. Trop. Med. Hyg..

[98] Maquart M., Boyer S., Rakotoharinome V.M., Ravaomanana J., Tantely M.L., Heraud J.M., Cardinale E. (2016). High prevalence of West Nile virus in domestic birds and detection in 2 new mosquito species in Madagascar. PLoS ONE.

[99] Venter M., Pretorius M., Fuller J.A., Botha E., Rakgotho M., Stivaktas V., Weyer C., Romito M., Williams J. (2017). West Nile virus lineage 2 in horses and other animals with neurologic disease, South Africa, 2008–2015. Emerg. Infect. Dis..

[100] Mostashari F., Bunning M.L., Kitsutani P.T., Singer D.A., Nash D., Cooper M.J., Katz N., Liljebjelke K.A., Biggerstaff B.J., Fine A.D. (2001). Epidemic West Nile encephalitis, New York, 1999: Results of a household-based seroepidemiological survey. Lancet.

[101] Komar N. (2003). West Nile virus: Epidemiology and ecology in North America. Adv. Virus Res..

[102] LaDeau S.L., Kilpatrick A.M., Marra P.P. (2007). West Nile virus emergence and large-scale declines of North American bird populations. Nature.

[103] CDC (Centers for Disease Control and Prevention) (1999). Outbreak of West Nile-like viral encephalitis—New York, 1999. Morb. Mortal. Wkly. Rep..

[104] Eidson M., Komar N., Sorhage F., Nelson R., Talbot T., Mostashari F., McLean R., West Nile Virus Avian Mortality Surveillance Group (2001). Crow deaths as a sentinel surveillance system for West Nile virus in the northeastern United States, 1999. Emerg. Infect. Dis..

[105] Nielsen C.F., Armijos M.V., Wheeler S., Carpenter T.E., Boyce W.M., Kelley K., Brown D., Scott T.W., Reisen W.K. (2008). Risk factors associated with human infection during the 2006 West Nile virus outbreak in Davis, a residential community in northern California. Am. J. Trop. Med. Hyg..

[106] Murray K.O., Mertens E., Desprès P. (2010). West Nile virus and its emergence in the United States of America. Vet. Res..

[107] Castro-Jorge L.A.D., Siconelli M.J.L., Ribeiro B.D.S., Moraes F.M.D., Moraes J.B.D., Agostinho M.R., Klein T.M., Floriano V.G., Fonseca B.A.L.D. (2019). West Nile virus infections are here! Are we prepared to face another flavivirus epidemic?. Rev. Soc. Bras. Med. Trop..

[108] Elizondo-Quiroga D., Elizondo-Quiroga A. (2013). West Nile virus and its theories, a big puzzle in Mexico and Latin America. J. Glob. Infect. Dis..

[109] Gubler D.J. (2007). The continuing spread of West Nile virus in the western hemisphere. Clin. Infect. Dis..

[110] Keesing F., Ostfeld R.S. (2021). Dilution effects in disease ecology. Ecol. Lett..

[111] ECDC (European Centre for Disease Prevention and Control) Factsheet About West Nile Virus Infection. https://www.ecdc.europa.eu/en/west-nile-fever/facts.

[112] Nagy A., Bán E., Nagy O., Ferenczi E., Farkas Á., Bányai K., Farkas S., Takács M. (2016). Detection and Sequencing of West Nile Virus RNA from Human Urine and Serum Samples during the 2014 Seasonal Period. Arch. Virol..

[113] Nath A., Tyler K.L. (2013). Novel Approaches and Challenges to Treatment of Central Nervous System Viral Infections. Ann. Neurol..

[114] Cui J., Zhao Y., Wang H., Qiu B., Cao Z., Li Q., Zhang Y., Yan F., Jin H., Wang T. (2016). Equine Immunoglobulin and Equine Neutralizing F(Ab′)2 Protect Mice from West Nile Virus Infection. Viruses.

[115] Gould C.V., Staples J.E., Huang C.Y.H., Brault A.C., Nett R.J. (2023). Combating West Nile Virus Disease—Time to Revisit Vaccination. N. Engl. J. Med..

[116] Tedesco C., Ruiz M., McLafferty S. (2010). Mosquito politics: Local vector control policies and the spread of West Nile Virus in the Chicago region. Health Place.

[117] Baz M.M., Selim A.M., Radwan I.T., Alkhaibari A.M., Gattan H.S., Alruhaili M.H., Alasmari S.M., Gad M.E. (2024). Evaluating Larvicidal, Ovicidal and Growth Inhibiting Activity of Five Medicinal Plant Extracts on *Culex Pipiens* (Diptera: Culicidae), the West Nile Virus Vector. Sci. Rep..

[118] EMCA (European Mosquito Control Association), WHO (World Health Organization) (2011). Guidelines for the Control of Mosquitoes of Public Health Importance in Europe. https://www.emca-online.eu/assets/PDFs/EMCA_WHOEURO-Guidelines_Control_Mosquitoes_PH_Importance_Europe-2013.pdf.

[119] Chaskopoulou A., L’Ambert G., Petric D., Bellini R., Zgomba M., Groen T.A., Marrama L., Bicout D.J. (2016). Ecology of West Nile virus across four European countries: Review of weather profiles, vector population dynamics and vector control response. Parasites Vectors.

[120] Bellini R., Zeller H., Van Bortel W. (2014). A review of the vector management methods to prevent and control outbreaks of West Nile virus infection and the challenge for Europe. Parasites Vectors.

[121] Fleischmann W.A., Nurjadi D., Velavan T.P. (2024). Addressing the rise of autochthonous vector-borne diseases in a warming Europe. Int. J. Infect. Dis..

[122] Odigie A.E., Stufano A., Schino V., Zarea A.A.K., Ndiana L.A., Mrenoshki D., Ugochukwu I.C.I., Lovreglio P., Greco G., Pratelli A. (2024). West Nile Virus Infection in Occupational Settings—A Systematic Review. Pathogens.

[123] Faverjon C., Vial F., Andersson M.G., Lecollinet S., Leblond A. (2016). Early Detection of West Nile Virus in France: Quantitative Assessment of Syndromic Surveillance System Using Nervous Signs in Horses. Epidemiol. Infect..

